# Developmental morphology of cover crop species exhibit contrasting behaviour to changes in soil bulk density, revealed by X-ray computed tomography

**DOI:** 10.1371/journal.pone.0181872

**Published:** 2017-07-28

**Authors:** Jasmine E. Burr-Hersey, Sacha J. Mooney, A. Glyn Bengough, Stefan Mairhofer, Karl Ritz

**Affiliations:** 1 Division of Agricultural & Environmental Science, School of Bioscience, University of Nottingham, Sutton Bonington Campus, Leicestershire, United Kingdom; 2 The James Hutton Institute, Invergowrie, Dundee, United Kingdom; 3 School of Science and Engineering, University of Dundee, Dundee, United Kingdom; 4 School of Computer Science, University of Nottingham, Nottingham, United Kingdom; University of Massachusetts Amherst, UNITED STATES

## Abstract

Plant roots growing through soil typically encounter considerable structural heterogeneity, and local variations in soil dry bulk density. The way the *in situ* architecture of root systems of different species respond to such heterogeneity is poorly understood due to challenges in visualising roots growing in soil. The objective of this study was to visualise and quantify the impact of abrupt changes in soil bulk density on the roots of three cover crop species with contrasting inherent root morphologies, *viz*. tillage radish (*Raphanus sativus*), vetch (*Vicia sativa*) and black oat (*Avena strigosa*). The species were grown in soil columns containing a two-layer compaction treatment featuring a 1.2 g cm^-3^ (uncompacted) zone overlaying a 1.4 g cm^-3^ (compacted) zone. Three-dimensional visualisations of the root architecture were generated via X-ray computed tomography, and an automated root-segmentation imaging algorithm. Three classes of behaviour were manifest as a result of roots encountering the compacted interface, directly related to the species. For radish, there was switch from a single tap-root to multiple perpendicular roots which penetrated the compacted zone, whilst for vetch primary roots were diverted more horizontally with limited lateral growth at less acute angles. Black oat roots penetrated the compacted zone with no apparent deviation. Smaller root volume, surface area and lateral growth were consistently observed in the compacted zone in comparison to the uncompacted zone across all species. The rapid transition in soil bulk density had a large effect on root morphology that differed greatly between species, with major implications for how these cover crops will modify and interact with soil structure.

## Introduction

Soil compaction is associated with soil physical stresses of mechanical impedance and hypoxia) that often impair the growth of crop plants [1]. Typically, within an agricultural context, the passage of wheeled and tracked machinery, the action of tillage equipment, and the trampling of animals produce compressive forces, which result in the compaction of the soil [2–4]. Compaction decreases the mean size of pores in the soil matrix, and can adversely affect root growth [5]. Roots are immersed in a soil matrix that has a range of inherent properties that affect their structural development, function and anatomy. Root development can be affected by a number of soil factors including texture, structure and degree of compaction [6]. The effect of such compaction can be physically restrictive, impairing the development of the root system and negatively affecting overall plant growth and crop yield [7, 8].

The variation observed between plant species in their capability to penetrate strong soil may be at least partly attributed to the tendency of roots to buckle or deflect, rather than to any inherent difference in root growth pressure or root elongation rate *per se* [9]. Roots typically follow indirect routes through the soil, seeking out the path of least resistance as they progress, and are able to do so due to their flexible nature [10]. Roots growing through soil experience mechanical impedance to varying degrees of severity. Often this can result in changes in the patterns of lateral root formation as well as considerably decreasing rates of root elongation and increasing root diameter [10, 11]. Roots may be unable to generate the forces required to displace soil particles to allow further extension into the soil profile [5]. Indeed, the strength of many UK arable soils is sufficient to substantially slow root elongation due to mechanical impedance, even when the soil is relatively wet (soil strength is the resistance of soil to deformation by an applied force) [12,13].

The use of plant roots as a tillage tool may offer a novel, practical solution to help remediate soil compaction. The creation of pores by deeply penetrating plant roots results in biologically-created pores, termed biopores. This process has also been termed ‘bio-drilling’, and allows the subsequent use of such biopores by the roots of succeeding crops as preferential pathways offering low resistance to growth [14]. In soils where crop plants suffer from high levels of mechanical impedance, this is considered to be beneficial [15]. In this context, cover crops (plants grown between harvested crops within rotations) provide a potential means of creating soil biopores which succeeding crop plants can utilise. Where the size of soil pores within the matrix are smaller than the diameter of the penetrating roots, plants species differ in their capability to penetrate the soil [9, 10]. The ability of roots to preferentially seek out biopores allows roots growing in compacted soil to negotiate otherwise impenetrable soil [16]. Roots clustering within biopores are common in compacted soils [17, 18] and may negatively affect the ability of roots to uptake water and nutrients [19]. Identifying plants that exhibit root architectural and morphological traits that enable them to penetrate soil to produce new pores, is key to determine species which could act as biodrilling cover crops.

Thick primary roots (possessed by many dicotyledonous species) have been shown to maintain faster relative root elongation rates compared to thin (monocotyledon) roots when grown in dense compressed sand [20], and also to penetrate compacted soils to greater depth in the field [21]. Tap-rooted species may exhibit better penetration abilities in compacted soils compared to fibrous rooted species and therefore be more beneficial for use in biological tillage [22]. These findings are also consistent with the use of thick-rooted dicotyledonous crops in rotations, as they may provide more effective penetration of compacted subsoil layers [23]. As well as having better penetration capabilities, crop species with taproots are capable of creating soil biopores >2 mm in diameter [15].

Many factors can affect root morphology and architecture, and it is clear that root systems have an innate plasticity to respond and adapt to their local environment [24]. The incorporation of species with a deep tap root system within crop rotations is desirable to help reduce the effects of soil compaction [25]. Roots of dicotyledonous species (notably lupin, faba bean and medic) thickened very substantially when grown in compressed sand, and had elongation rates that were relatively less impeded than monocotyledons that thickened less [21]. However, these particular experiments were performed at very large mechanical impedances (sufficient to slow root elongation by 88–97%). Maize plants grown in compacted soil were shown to have fewer lateral roots than plants grown under control conditions [26].

Although there are many studies on the response of crop plants to soil compaction [e.g. 22, 25, 27, 28], relatively few have examined the influence of bulk density p*er se* on root system architecture and response in 3D [11]. Dry bulk density (generally referred to as bulk density in this manuscript) describes the mass of oven-dried soil in a given volume, and generally becomes greater with an increase in compaction pressures and soil depth [29]. A recent study [30] looked at the ability of root tips to deform soil of a low bulk density in 4D, however detailed studies on the effect of compaction on root system architecture of cover crops are lacking. Previous field studies indicated tillage radish had more than twice as many roots per unit volume as rye, and that root counts at depth actually increased for tillage radish with increasing soil strength [22]. The mechanism for this increase in root number was not clear, although the authors hypothesise that it was associated with a substantial change in root system morphology. The development of non-destructive measurement techniques for imaging root architecture *in situ*, makes it possible to determine the geometric properties of root systems [31] which are essential to understand their growth dynamics [32].

The objective of this study was to assess the effects of a sharp transition in bulk density on the root system architecture of three contrasting cover crop species (tillage radish, vetch, and black oats) *in situ*. X-ray computed tomography was used to study root system morphology and trajectory responses to the presence of the compacted layer, with regard to the potential effectiveness of these species for applications in compaction alleviation via bio-drilling.

## Materials and methods

### Sample preparation

A Dunnington Heath series sandy loam (66.4% sand 18% silt, 15.6% clay) was obtained from the University of Nottingham experimental farm at Sutton Bonington, Leicestershire, UK (52°50’07”N, 1°15’04.0”W). The soil was air dried and sieved to <2 mm. Columns (160 mm height x 75 mm diameter) were uniformly packed in a two phase system to provide dry bulk densities of 1.2 g cm^-3^ (uncompacted) overlaying 1.4 g cm^-3^ (compacted). Penetrometer resistance readings were 0.21 (± 0.01) MPa and 0.30 (± 0.03) MPa respectively for the two bulk density layers. These were packed with air-dried soil in *circa* 3 cm deep layers (bottom 10 cm compacted to 1.4 g cm^-3^ / top 6 cm compacted to 1.2 g cm^-3^). After compacting each layer the surface was lightly scarified to ensure homogeneous packing [33]. Four replicates were prepared for each species plus unplanted controls (originally allowing for two destructive harvests) to give a total of 28 columns. The columns were then saturated from below for 48 h and allowed to gravimetrically drain to a notional field capacity of approximately 21%. The air-filled porosity at field capacity will have been greater than 20% in both the uncompacted and compacted layers.

Seeds of tillage radish (*Raphanus sativus* L. cv. Early Mino), vetch (*Vicia sativa* L. cv. Buza) and black oat (*Avena strigosa* Schreb cv. Pratex) procured from Frontier Agriculture Ltd, Norfolk, UK, were soaked in water for 12 h before planting. Three seeds of each species were sown in each of 8 replicate columns approximately 1.5 cm below the surface. Once the shoots emerged they were thinned to one plant per column. Columns were placed in a glasshouse in a random block design (four blocks featuring one replicate of each species x harvest; average day/night temperature of 24°/ 18°C) and were watered daily with a nutrient spiked (HortiMix Standard: 15-7-30 +1.6MgO + TE (2: 1: 4)) water solution.

### Image processing and analysis

The four unplanted control columns were CT scanned on Day 0. A subset of 12 columns featuring the three species with four replicates was scanned after 20 days and a second subset of 12 columns was scanned after 58 days. Prior to imaging the soil columns were not watered for a period of 48 h to ensure a high level of contrast between soil, organic matter and root material. All columns were scanned using a Phoenix V|Tome |X m X-ray 240kV (GE Measurement & Control Solutions, Wunstorf, Germany) at the Hounsfield Facility at the University of Nottingham, with a 0.1 mm copper filter. Each projection image was an integration of 3 images with a skip setting of 1 discarded image. Voxel resolution was set at 60 μm, potential energy of 180 kV and current of 180 uA. Columns were scanned in two sections with a total scan time of 75 mins per column. The distance from the sample to the source was 245 mm. A total of 2160 image projections were captured for each column. Images were reconstructed at 32-bit using Phoenix Datosx 2 reconstruction tool to form a 3D volume which was visualised using VG Studio Max 2.2.5 software.

An automated image processing algorithm, ‘RooTrak’, was used to segment roots from the μCT grey scale images [34]. The method employs a tracking-based strategy which follows the root cross-sections through a sequence of images, with the initial digital processing seed-point being identified manually by the user. As the image stack is traversed, the cross-sections move around the image, reproducing the shape of the scanned root. The tolerance values were adjusted following visual inspection to ensure only root material was included in the region of interest (ROI) produced. Once the root system was successfully extracted from the μCT images it was transferred as a bitmap (.bmp) image stack to VG Studio MAX v. 2.2.5 for editing. The finalised volumetric representation of the root system architecture was analysed to quantify both global and local traits in RooTrak v. 0.3.9.1 [35]. Global traits derived from the entire root system were volume (%), surface area (cm^2^), maximum depth (mm), maximum width (mm), convex hull (cm^3^) and solidity (mm^3^). Local traits were derived from the individual roots or a topical zone of interest and included the number of lateral roots protruding from the primary root (lateral number) and the angle at which they projected (lateral angle), which were subsequently calculated using VG Studio Max. Global traits were considered for the root systems as a whole for all species. A selection of traits including root volume (%), surface area (mm^2^), number and angle of lateral roots were calculated for the uncompacted and compacted zones within the column by splitting the image stacks at the transition zone between the different bulk densities. This allowed a layer comparison over time to be conducted on the individual species.

The root volume provides an estimate of the sum of the cross-sectional areas for all of the voxels in the root system and the total mass of the root system [36], here calculated as a percentage volume of the total soil volume. Root surface area was calculated by summing the representative isosurface of the segmented root system to provide an approximate surface area of root that is in direct contact with the soil [34]. Root depth measurement was calculated as the maximum vertical distance reached by the root system in relation to the first image slice containing a root system voxel [24]. Root system width was calculated as the maximum horizontal width of the whole root system architecture. The convex hull was determined as the smallest convex set of voxels that contain the root system and indicates the effective volume of soil explored by the root system, and allows a formalised geometric analysis of the zone of influence of the root system [35]. Solidity was calculated as the fraction equal to the root volume divided by the convex hull volume in 3D, and partly indicates the relative spatial ability of roots to obtain water and nutrients. Lateral number was determined by a manual count of the lateral roots protruding from the primary root using the indicator tool in VG Studio Max. Lateral initiation angle was measured using the 3-point angle tool in VG Studio Max and describes the angle of the root at its join to the primary root; an angle of 0 degrees indicates vertical growth and 90 degrees indicates horizontal growth. Once the root systems had been analysed using RooTrak the output image stacks were imported into Blender 2.77 (open source) for image rendering.

The CT resolved air filled pore space (porosity) of the columns was calculated using ImageJ-win64. Due to the resolution of the images in this study only the air filled pore space was included in the soil porosity measurements. This is because the grey scale colouration of water filled pores and organic matter is extremely similar. By discounting water filled pores, porosity measurement is not over-estimated for the scale of the imagery by including unwanted organic matter. Two image stacks of 500 grey scale image slices were extracted separately for the two bulk density layers in each column and the porosity for each sample cube was averaged. A macro was used to automatically crop, enhance brightness/ contrast, apply median filter, radius 1, apply ‘minerror’ threshold and convert data to 8-bit. Two sample sites were selected with different start coordinates in each layer and an area of 27 cm^3^ was analysed.

To establish whether the time, compaction or species effect had interactions as explanatory variables on the global and local root traits, a two-way factorial analysis of variance (ANOVA) with specified block design was undertaken on the columns as a full system comparing all species and on the two bulk density layers comparing species individually. Soil porosity values were analysed using ANOVA allowing for split plot design for bulk density layers within the cores. Statistical tests were conducted using Genstat 17th edition and included requisite tests for distribution and homogeneity of variance. Treatment effects were considered significant when p < 0.05.

## Results

### Whole root architecture analysis

**Fig 1:**
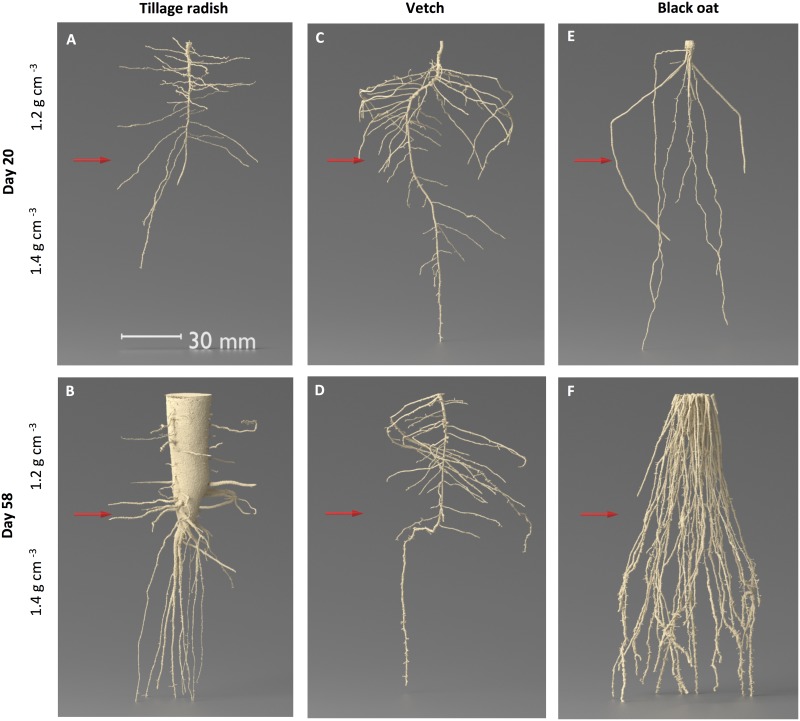
Representative 3D renderings of root systems grown in repacked soil columns. (A) tillage radish after 20 days growth; (B) tillage radish after 58 days growth; (C) vetch after 20 days growth; (D) vetch after 58 days growth; (E) black oat after 20 days growth; (F) black oat after 58 days growth. Red arrows indicate location of change in dry bulk density of soil profile from 1.2 to 1.4 g cm^-3^.

**Fig 2:**
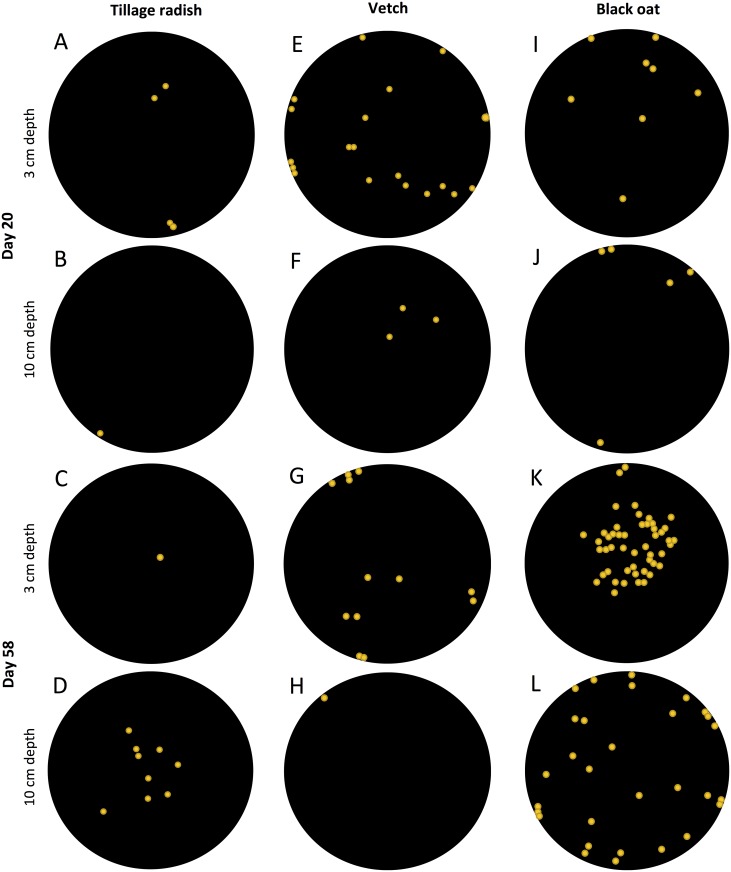
Location maps of roots in 2D cross-section representative slices of segmented root systems shown in Fig 1. (A-D) Tillage radish: (A-B)) Day 20; (C-D) Day 58; (E-H) Vetch: (E-F) Day 20; (G-H) Day 58; (I-L) Black oat: (I-J) Day 20; (K-L) Day 58. Orange circles denote position of root within column and do not relate to root diameter (actual sizes would be indiscernible in many cases at this scale of imaging; diameter of core, here black circles, is 75 mm). For all species 3 cm depth falls within the uncompacted zone and 10 cm depth is within the uncompacted zone.

Whole three-dimensional visualisations of the three species’ root systems grown within a two-phase compaction treatment were analysed based on renderings of X-ray CT data. They revealed a marked contrast in the overall root system architecture between the three species, and a substantial effect of soil bulk density on root behaviour was observed (Figs 1 and 2). Tillage radish developed a taproot system with extensive lateral roots by 20 days with substantial radial tap root expansion by 58 days, entirely confined to the uncompacted zone, and a series of vertically-penetrating finer roots arising from the terminus of the tap root into the compacted zone (Fig 1A and 1B). Vetch produced a diffuse primary root system largely confined to the uncompacted zone, with very little penetration of the compacted zone and little change between Days 20 and 58 (Fig 1C and 1D). The root system frequently exhibited a deflection of the taproot towards the column wall upon encountering the compacted zone (Fig 1D). Black oat developed mainly vertically-oriented primary roots which were very prolific in the uncompacted zone, but also penetrated the compacted zone, increasing in frequency between Days 20 and 58 (Fig 1E and 1F). There was no evidence for any vertically upward growth of roots as a result of encountering the interface between the bulk density phases for any of the species.

**Fig 3:**
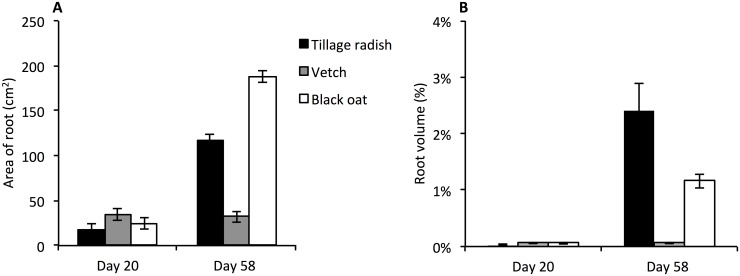
Average surface area of root (cm^2^) and root volume as% of full soil column. (A) Surface area of root (cm^2^) within full soil column for tillage radish, vetch and black oat root systems, derived from X-ray CT data. Bars denote means, whiskers pooled SE. (B) Volume of root as a percentage of the uncompacted and compacted soil zones for tillage radish, vetch and black oat root systems, derived from X-ray CT data. Due to the disproportionately large increase in total root volume for tillage radish the data was not subject to ANOVA. Bars denote means, whiskers individual standard deviation.

In all cases, roots that made contact with the edges of the columns subsequently tracked these boundaries by growing vertically downwards (Figs 1 and 2). Whilst at Day 20 roots of tillage radish and black oat were predominantly located in this peripheral region (Fig 2B and 2J), by Day 58 newly formed roots showed little lateral deviation as a result of entering the compacted zone and were subsequently prolific within this layer (Fig 2D and 2L). Penetration of the compacted zone by vetch was variable between replicates and in most cases roots were confined to the peripheral zone adjacent to the column wall. Where vetch roots penetrated the compacted zone, they tended to grow laterally (Figs 1C and 2F). Rapid vertical growth was observed with all species penetrating the compacted zone by Day 20, at Day 58 the full depth of the columns had been explored by all root systems. At Day 20 the total surface area of root systems was similar between the three species, increasing significantly between Days 20 and 58 for tillage radish and black oat, but not for vetch (Fig 3; species x time interaction p = < 0.001, n = 24). Similar trends were observed in the root volume data (Fig 3B), with tillage radish producing a notably large volume as a result of radial expansion of the tap-root. The relative total volume for tillage radish roots was 48 times greater than for vetch roots, and twice that of black oat roots. Relative total volumes in the compacted zone revealed that mean tillage radish root volumes were 7.3 times greater than vetch root volumes and 1.2 times greater than black oat.

**Fig 4:**
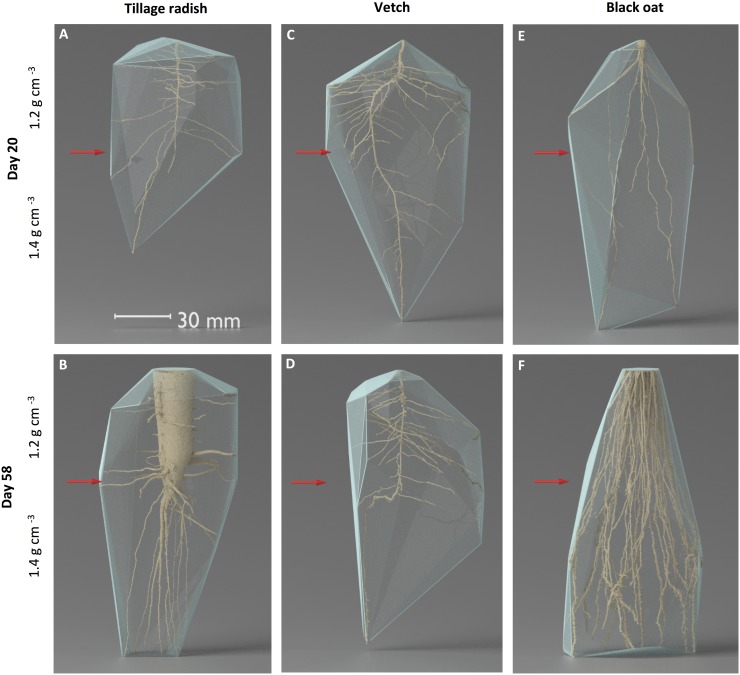
Visual representation of convex hull volumes for representative root systems, red arrows indicate change in dry bulk density of soil profile. (A) Tillage radish after 20 days growth. (B) Vetch after 20 days growth. (C) Black oat after 20 days growth. (D) Tillage radish after 58 days growth. (E) Vetch after 58 days growth. (F) Black oat after 58 days growth.

**Fig 5:**
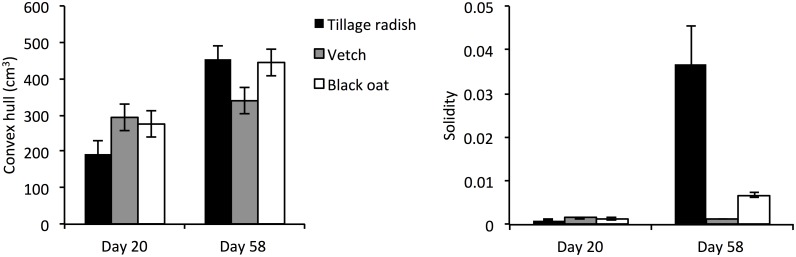
Average root systems convex hull (cm^3^) and solidity value of full soil column. (A) Convex hull (cm^3^) of tillage radish, vetch and black oat root systems. Data represents full root systems, derived from X-ray CT. Bars denote means, whiskers pooled SE. (B) Solidity values of tillage radish, vetch and black oat root systems. Data represents full root systems, derived from X-ray CT data. Due to extreme range of values, tillage radish the data was not subject to ANOVA. Bars denote means, whiskers standard error.

At Day 20 both tillage radish and vetch plants exhibited extensive lateral proliferation in the uncompacted zone resulting in a wide convex hull tapering into a thin angular hull in the compacted zone as the number of laterals decreased with depth (Fig 4A and 4B). Black oat roots depicted a thinner convex hull due to the vertical nature of root growth through both the uncompacted and compacted zones (Fig 4C). At Day 58 the convex hull volume of tillage radish revealed that the lateral proliferation decreased resulting in a thinner convex hull in the uncompacted zone, observed as a split in the taproot morphology into numerous vertical growing secondary roots, increasing the volume of the hull in the compacted zone (Fig 4B). The convex hull of the vetch roots at Day 58 was unchanged from Day 20 (Fig 4E). The convex hull of black oats exhibited a thin geometric shape in the lower layer spreading to a wide hull in the compacted zone as the roots proliferated towards the column walls and started to track down the periphery of the column (Fig 4F). Mean convex hull volumes increased significantly over time for tillage radish and black oat plants, whilst they were equal in vetch plants (Fig 5A; species x time interaction p = 0.031, n = 24). Likewise, solidity values increased over time for tillage radish and black oat, but not for vetch (Fig 5B).

### Uncompact versus compact comparison

#### Tillage radish

**Fig 6:**
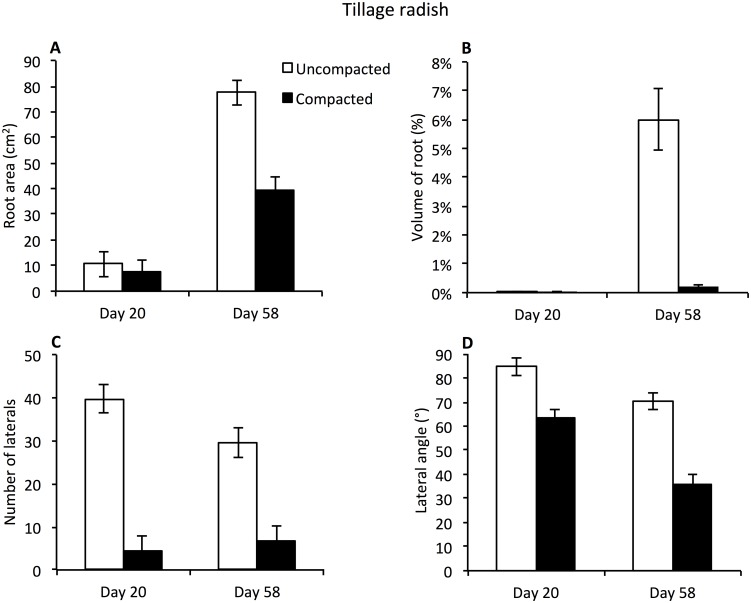
Average surface area (cm^2^), volume of root (%), number of laterals and lateral angle (°) of tillage radish root systems. (A) Average surface area of tillage radish root (cm^2^). (B) Average volume of tillage radish root as a percentage of the soil volumes. Due to extreme range of data values data was not subject to ANOVA. (C) Average number of lateral roots observed protruding from the taproot. (D) Average angle of lateral roots protruding from taproot. For all graphs data is presented for both the compacted and uncompacted zones over two time periods. Bars denote means, whiskers pooled (A, C, D) or individual (B) s.e.

Root surface area was similar at Day 20 in both the uncompacted and compacted zones. This increased significantly with time, significantly more so in the uncompacted zone, with 50% greater surface area in comparison to the compacted zone at Day 58 (Fig 6A; time x compaction interaction p = 0.004, n = 16). Root volume of tillage radish increased substantially as a result of tap root expansion by Day 58, which was exclusively confined to the uncompacted zone (Fig 6B). Whilst roots subsequently penetrated the compacted zone, their volume was some 30 times smaller than the tap root (Fig 6B). Greater numbers of lateral roots were observed in the uncompacted zone compared to the compacted, with means of 35 and 6 respectively (compaction effect p = <0.001, n = 16; Fig 6C). The number of lateral roots between the uncompacted and compacted zones did not change over time (Fig 6C). Only main effects in ANOVA were apparent with respect to the branch angle of lateral roots (Fig 6A). These decreased significantly in the compacted zone in comparison to the uncompacted zone with overall means of 75° and 50° respectively (p = <0.001, n = 16). Branch angle also decreased over time in both the uncompacted and compacted zones (overall means 77° and 49° respectively p<0.001, n = 16).

#### Vetch

**Fig 7:**
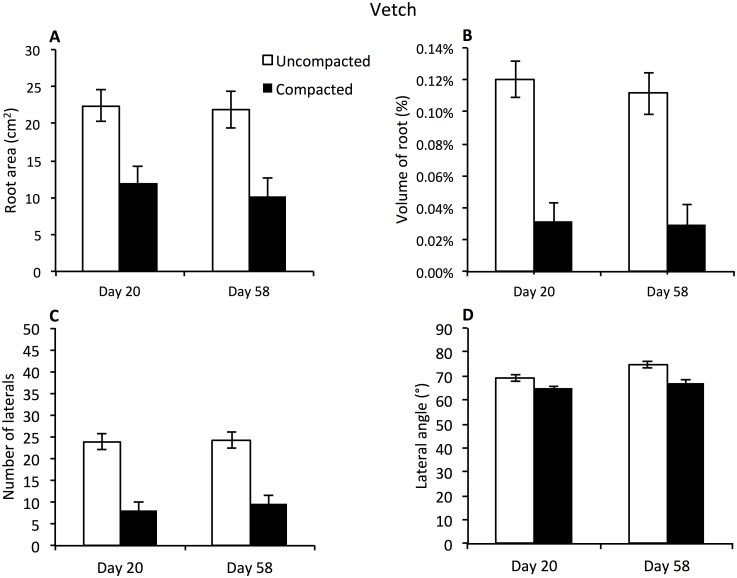
Average surface area (cm^2^), volume of root (%), number of laterals and lateral angle (°) of vetch root systems. (A) Average surface area of vetch root (cm^2^) (B) Average volume of vetch root as a percentage of the soil volumes, (C) Average number of lateral roots observed protruding from the taproot into the soil volumes. (D) Average angle of lateral roots protruding from taproot into the soil volumes. For all graphs data is presented for both the compacted and uncompacted zones over two time periods Bars denote means, whiskers pooled SE.

For vetch, there was a significant effect of compaction on root surface area, with overall means of 110 cm^2^ and 221 cm^2^ for compacted and uncompacted respectively (p<0.001, n = 16; Fig 7A) and hence no significant change over time. This phenomenon was mirrored in the root volume data with a difference of 0.09% between uncompacted and compacted zones (Fig 7B; compaction effect p = <0.001, n = 16). A 70% reduction in number of lateral roots was observed in the compacted zone compared to the uncompacted zone at both Days 20 and 58 (Fig 7C; compaction effect p < .001, n = 16). Branch angle was observed to decrease in the compacted zone by 5° (Fig 7D; compaction effect p = 0.002, n = 16).

#### Black oat

**Fig 8:**
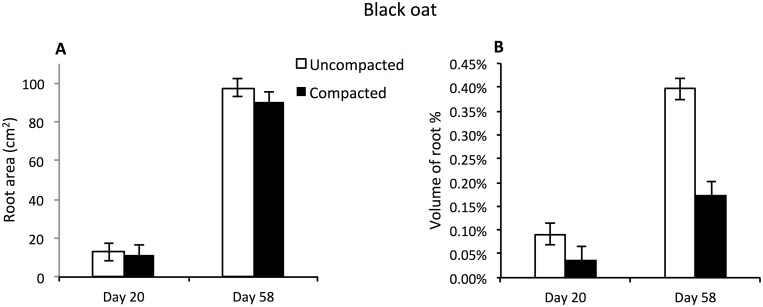
Average surface area (cm^2^), volume of root (%) of black oat root systems. (A) Average surface area of black oat root (cm^2^). (B) Average volume of black oat root as a percentage of the soil volume. For all graphs data is presented for both the compacted and uncompacted zones over two time periods. Bars denote means, whiskers s.e.

Root surface area of oats increased significantly between Days 20 and 58 (123 cm^2^ and 939 cm^2^ respectively; p<0.001, n = 16), with no effect of compaction or interaction over time (Fig 8A). An increase in root volume was observed in both the uncompacted and compacted zones over time, with a substantial volume increase of 0.3% in the uncompacted zone (Fig 8B; time x compaction interaction p = 0.006, n = 16).

### Soil porosity

**Fig 9:**
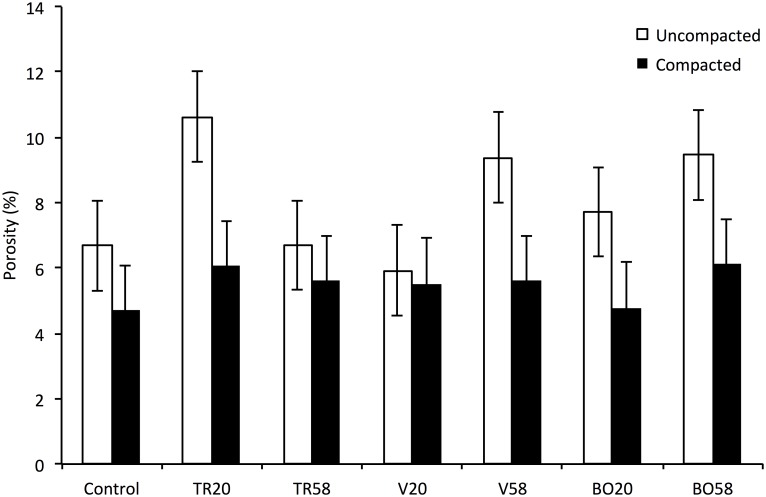
Soil porosity of uncompacted and compacted layers with tillage radish, vetch and black oat roots systems growing at three time points. Average percentage of pore space from representative samples within both the uncompacted and compacted soil volumes, bars denote means, whiskers pooled s.e.

A marginally significant third-order interaction between species, compaction and time was observed in relation to soil porosity (measured at a scale of 60 μm) (p = 0.058, n = 56; Fig 9). Porosity was of the range 5–6% in the compacted layer, compared to 7–10% in the uncompacted layer. No significant difference in porosity between control columns and vetch or black oat plants was observed in the uncompacted zone at Day 20. However there was a significant difference indicating an increase in porosity between control columns and tillage radish columns at Day 20 in the uncompacted zone. No significant difference in porosity was observed in the compacted zone in any species at either Day 20 or Day 58 in comparison to Day 0. Black oat plants exhibited a significant difference in porosity between the uncompacted and compacted zones at both Day 20 and Day 58. In vetch this significant effect was only exhibited by plants at Day 58, and in tillage radish plants at Day 20.

## Discussion

Soil dry bulk densities typically encountered in the field can range from between 1.1 g cm ^-3^ to 1.6 g cm^-3^ with the denser soils restricting plant root development [36]. This study mimicked bulk density transitions which may occur frequently in the field, often as 'plough pans', manifest as a sharp increase in bulk density across a plane at the plough depth (typically *circa* 20 cm), but also more sporadically as a result of general trafficking or soil structural collapse after rainfall [4]. The geometry and scale of bulk density transitions will vary in the field. In our experimental system, the scale of the design was prescribed to optimise the trade-off between the spatial resolution of the CT scanning (smaller volumes providing greater resolution) and a volume of soil that would allow a reasonable development of the plants, whilst providing the key feature of the presence of an interface between relatively low and high soil bulk densities.

The restriction of root growth within compacted soils depends on the species and the age of the plants [1, 22], which is why plants were imaged at both juvenile and more mature growth stages. The roots grown in this experiment will have experienced a decrease in aeration and a small increase in soil penetration resistance (0.21 to 0.30 MPa), and increase in root-soil contact with depth as they enter the more compacted zone. These three changes in soil aeration, soil strength, and root-soil contact represent relatively benign soil physical conditions that would not normally be expected to be a major restriction to root elongation.

At Day 20 both vetch and black oat plants had a mean root volume of 0.06% in comparison to tillage radish 0.03%. Despite having the same mean volume as black oat, vetch plants had the greatest mean solidity, convex hull volume and root surface area values, indicating that at the juvenile growth stage vetch had the greatest ratio between the volume of the column explored in comparison to the volume of the root system. The solidity of the root system is a functionally important indicator in root foraging, and the higher its relative value, the greater the root systems spatial suitability to obtain water and nutrients [37]. Hence at Day 20 the imaged roots of the vetch plants had explored the soil column much better than those of the other two species. Despite this, porosity data indicated that tillage radish plants at Day 20 had significantly increased the air filled porosity within the uncompacted zone. Unlike the other two species, this increase may be attributed to high volumes of fine root mass not accounted for in the 60 μm resolution of the scans. At Day 58 tillage radish root systems exhibited the greatest mean solidity, root volume and convex hull volume; however, in this case of the tillage radish taproot in the upper layer, the bulk of the root volume was contained in one very thick taproot—and so would be relatively ineffective at extracting soil resources as compared with a more finely divided root system—indicating that the concept of solidity cannot be used in isolation. The presence of this large root was reflected in the porosity measurement with tillage radish plants at Day 58 showing a decrease in porosity due to a large volume of the uncompacted zone being comprised by the taproot. The large root system of the tillage radish was mainly concentrated in the tap root, as compared with the volume of black oat root systems that was widely dispersed by more individual roots within the compacted zone. Under field conditions the ability of roots of various crops to penetrate compacted soils is a reflection of their relative capacity to produce roots under such compacted conditions [21]. The juvenile vetch plants initially produced root volume fastest but, by the mature growth stages, both tillage radish and oat had overtaken the vetch. However, the roots that penetrated into the compacted zone did not significantly increase the porosity of the soil at a resolution of 60 μm.

A number of studies (e.g. [21, 22]) suggest that dicot roots are better able to penetrate compacted subsoil in the field than monocot roots, and species with thicker roots tend to exhibit greater penetration of the subsoil. For tillage radish it was not the thicker roots *per se* which penetrated the compacted layer, but instead the root system architecture response was for the single thick taproot to branch into multiple finer roots (‘fanging’; [38]), which penetrated the denser soil. In preliminary studies, when tillage radish was grown in a soil of uniform bulk density, no fanging occurred at depths of up to 83 cm (S1 Fig). Hence it appears to be the transition in bulk density that triggered the morphological changes observed. Similar fanging-style morphological responses of sugar beet and carrots to mechanical impedance has also been observed in compacted field soils [39]. As a result, for tillage radish there was a proliferation of roots within the compacted zone which then grew at an acute angle downward, with no evidence for tracking a path of least resistance towards to edge of the column.

Lateral roots are generally finer than a parent taproot and can penetrate smaller pores [9]. The observed fanging of the tillage radish may increase the exploratory capabilities of the root system. In the field subsoil pans tend to decrease rooting depth, this is commonly associated with an increase in root biomass in the topsoil as a result of increased lateral formation by those roots experiencing impedance at their apices. The thickening of primary roots and increased proliferation of lateral roots in the loose soil located above a high-strength soil layer has been previously reported [40]. It is likely that increased levels of ethylene produced by stressed roots in compacted soil and subsequent higher availability of carbohydrates due to impeded translocation of substrate from the shoot when the growth of the main axis is restricted may results in the increased proliferation of lateral roots above a homogeneous compacted layer [22]. The images of the root systems for vetch and tillage radish at seem at least qualitatively consistent with the concept of increased length of laterals in the upper (uncompacted) layer, although the finest roots will not have been resolved by the CT due to the spatial limit of detection.

A change in tap root morphology at the interface between the two bulk density phases of the vetch plants was observed in the majority of cases; the primary root growth angle was deflected at the compacted phase change to follow the path of least resistance horizontally across the soil profile to the column wall. Roots may not be able to penetrate the subsoil from weaker surface layers if they are very flexible [20]. The trajectories of root tip growth differed markedly between the three species on encountering even the modest change in soil strength at the interface between the layers. Vetch was most sensitive with its root tip diverting from the vertical to the greatest extent, tillage radish exhibited marked fanging of the taproot, whilst black oat roots were not noticeably diverted. We are not aware that such large differences between species in this characteristic has been reported previously.

Despite tillage radish having the greatest mean root volume in the compacted zone, black oat exhibited the greatest mean root surface area, because the root volume consisted of many thinner roots. A greater root surface area means a higher degree of root:soil surface contact which may be beneficial for nutrient and water uptake [41]. As a result more roots penetrated directly through the compacted zone in black oat plants, indicating the trajectory of root growth was least affected by the bulk density change. The more roots that can penetrate a soil mass, the greater the potential improvement to the soil through changes in soil pore size distribution [21]. In the case of the species studied, the greater number of roots which were able to penetrate into the compacted zone, the greater the potential rate of soil amelioration. Both tillage radish and black oat exhibited evidence of roots penetrating the compacted zone without the aid of the column wall, suggesting that in field realistic conditions they are more likely to have the ability to grow vertically through a compacted soil profile as opposed to deflecting and resulting in high volumes of root in the topsoil. From the data collected at the mature growth stage, both tillage radish and black oat provide contrasting root system architectures each with the ability to consistently penetrate a compacted zone. Although the porosity of the lower soil layer did not change during this study, the ultimate decay of the roots would leave biopore channels in the soil that subsequent crop roots might exploit. Both tillage radish and black oat may therefore be favourable candidate species for compaction alleviation via bio-drilling.

The three modes of morphological response exhibited by the different species here are complementary in terms of the way the soil volumes in the two soil bulk density layers are exploited, particularly after 58 days. This is apparent in 2-D visualisations, where combined root distributions in both uncompacted (Fig 2C, 2G and 2K) and compacted (Fig 2D, 2H and 2L) zones collectively span the entire plane; and in 3-D visualisations, where the convex hull shells have a highly complementary geometry (Fig 4B, 4E and 4F). These morphological types have here been manifest when the plants are grown in isolation, and it remains to be seen if such trait complementarity would prevail when the plants are grown in combination—and in competition. Were it so, this would offer the potential that such mixtures could be adopted in circumstances where thorough exploration of the soil resource is required. The species prescribed for this study are commonly adopted as cover crops in arable rotations [22, 42, 43], and our study provides evidence as to why tillage radish and black oats in particular are likely effective species in this role where there is soil compaction. It also suggests that a mixture of the three might be more potent, particularly in terms of increasing the porosity of a soil via complementary penetrating and proliferating growth modes.

This study has provided a detailed 3D visualisation using a specialised automated image segmentation method to visualise the impact a change in bulk density of a sandy loam soil had on the root system architecture of three major cover crop species using non-destructive X-ray CT scanning. The spatial resolution of objects imaged by X-ray CT is prescribed by the total volume of the sample being scanned. Here, the limit of resolution was 60 μm and as such the roots visualised were confined to those greater than this dimension. Very fine root mass was not detected and therefore not considered in this study. The size of sample column restricted the maximum rooting width/ depth, convex hull and solidity measurements as the maximum volume of the sample was limited.

## Conclusion

Here we revealed the ontogeny of tillage radish, vetch and black oat root formation in 3D and provide insight into changes in root system architecture, specifically changes in taproot morphology and lateral root growth angles upon encountering a compacted soil layer. Smaller root volume, surface area, number of lateral roots and angle values were consistently observed in the compacted zone in comparison to the uncompacted across all species types. This suggests that plant roots can be sensitive to even a relatively small change in bulk density, which can directly inhibit growth and instigate changes in root morphology, particularly in tap-root species. Tillage radish root systems produced the greatest values at the mature growth stage for root volume, convex hull volume, solidity, lateral number and lateral angle. Black oat root systems produced the greatest root surface area at the mature growth stage and also had the greatest root proliferation within the compacted zone coupled with the least notable change in root morphology. Tillage radish and black oat root systems both appeared better suited for soil structural remediation.

## Supporting information

S1 Appendix Supplementary data, file contains soil porosity and root characteristic measurements for the 28 columns. (XLSX)

S1 Fig Tillage radish plants, *(Raphanus sativus)* grown in soil columns with dimensions of 85 cm depth, 17 cm width, packed to a bulk density of 1.5 g cm-3 with a soil mixture of Broughton clay loam soil 70%, Tunstall silver sand 30%, for 65 days. (TIFF)
