# Correction: Developmental morphology of cover crop species exhibit contrasting behaviour to changes in soil bulk density, revealed by X-ray computed tomography

**DOI:** 10.1371/journal.pone.0190759

**Published:** 2018-01-02

**Authors:** Jasmine E. Burr-Hersey, Sacha J. Mooney, A. Glyn Bengough, Stefan Mairhofer, Karl Ritz

There are errors in the Funding section. The correct funding information is as follows: This work was supported by the Biotechnology and Biological Sciences Research Council; and the Natural Environment Research Council [Grant number NE/M009106/1], by a Soils Training and Research Studentships (STARS) grant to JB-H. STARS is a consortium consisting of Bangor University, British Geological Survey, Centre for Ecology and Hydrology, Cranfield University, James Hutton Institute, Lancaster University, Rothamsted Research and the University of Nottingham. The James Hutton Institute receives funding from the Scottish Government.
