# Supplementary figures and images for: Developmental morphology of cover crop species exhibit contrasting behaviour to changes in soil bulk density, revealed by X-ray computed tomography
Source: PLoS One. 2017 Jul 28;12(7):e0181872. doi: 10.1371/journal.pone.0181872 (PMC5533331; doi:10.1371/journal.pone.0181872)

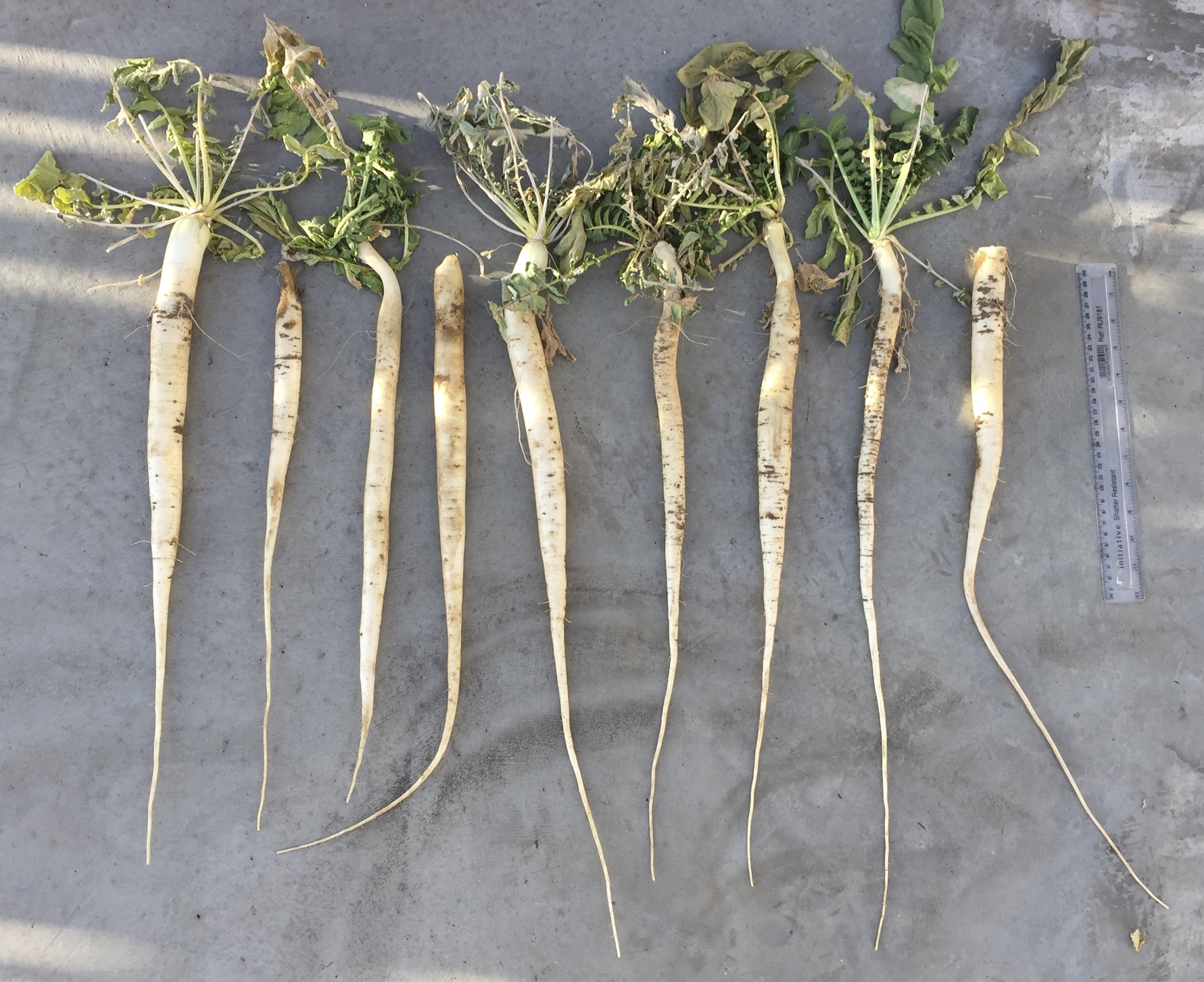

Supplement: S1 Fig — (TIFF) [file pone.0181872.s002.tiff]
